# Through the haze: a multinational cross-sectional comparison of cannabis risk knowledge gaps among young adults

**DOI:** 10.1186/s42238-026-00439-3

**Published:** 2026-04-28

**Authors:** Anne Maiwald, Elena Gomes de Matos, Sally Olderbak, Larissa Schwarzkopf, Gabriele Koller, Reiner Hanewinkel, David Hammond, Eva Hoch

**Affiliations:** 1 Department of Psychiatry and Psychotherapy, LMU University Hospital, LMU Munich, Munich, Germany; 2 IFT Institut für Therapieforschung, Centre for Mental Health and Addiction Research, Munich, Germany; 3 Division of Clinical Psychology and Psychotherapy, Charlotte Fresenius University, Munich, Germany; 4 Institute of Medical Information Processing, Biometry and Epidemiology (IBE), Faculty of Medicine, LMU Munich, Munich, Germany; 5 Faculty for Applied Healthcare Sciences, Technical University of Deggendorf, Deggendorf, Germany; 6 Institut für Therapie- und Gesundheitsforschung, IFT-Nord, Kiel, Germany; 7 School of Public Health Sciences, University of Waterloo, Waterloo, ON Canada

**Keywords:** Cannabis use, Risk knowledge, Young adults, Prevention

## Abstract

**Background:**

Young adults report heightened cannabis use yet show gaps in cannabis-related risk knowledge. Risk knowledge gaps in young adults with diverse cannabis use experience and across countries with varying cannabis policies, as well as associations with knowledge levels were explored.

**Methods:**

The International Cannabis Policy Study (ICPS) is a cross-sectional, web-based survey that uses non-probability sampling and post-stratification weighting. Data from the 2023 ICPS national surveys conducted in Canada, Germany and the UK were used. A total of *n* = 2,945 18- to 25-year-olds were included in the analyses (Canada: *n* = 2,047; Germany: *n* = 446; UK: *n* = 452). Risk knowledge gaps were assessed through 7 health-related risk items. Inaccurate responses were turned into an index variable to measure participants’ level of risk knowledge. Negative binomial regression models were used to examine associations between sociodemographic and use-related variables and knowledge level.

**Results:**

Risk knowledge levels were highest among German participants, and lowest among UK participants. Some risks were better known than others across all countries. Risk knowledge was lowest among regular cannabis consumers, for whom being from the UK was associated with decreased knowledge levels (IRR = 1.227) and being at moderate risk of harm from use was associated with increased knowledge levels (IRR = 0.701). Among occasional consumers, age (IRR = 1.041), being male (IRR = 1.222) and being at moderate (IRR = 1.236) and high (IRR = 1.818) risk of harm from use were associated with decreased risk knowledge, whereas peer use (IRR = 0.718) was associated with increased risk knowledge.

**Conclusions:**

This study showed that there are differences in the perception of cannabis risks among young adults, which are associated with individual consumption patterns as well as country-specific and sociodemographic factors. Findings extend the current understanding of differences and similarities in risk knowledge gaps among young adults across different countries, allowing for a more tailored risk education towards the needs of this target group.

## Introduction

Cannabis is the most widely used drug under the control of the (UN Convention on Narcotic Drugs 1961; Lande 1962) with an estimated 244 million people using in 2023 (United Nations Office on Drugs and Crime, 2025). Compared to other age groups, young adults report the highest levels of cannabis use, with past 12-month prevalence in 2023 estimated at 23.5% in Germany (18- to 25-year-olds, Orth et al. 2025), 13.8% in the United Kingdom (UK, 16- to 24-year-olds, Office for National Statistics, 2024) and 43% − 48% in Canada (16 to 24-year-olds, Health Canada, 2025). Early onset and a high frequency of use below the age of 25 both pose an increased risk of adverse health outcomes, such as cannabis use disorder, decline of bodily and cognitive function, and mental health issues (Connor et al. 2021; Hoch et al., 2024). This makes young people particularly vulnerable to long-term health impairment.

Heightened cannabis use levels among young adults have been linked to lowered cannabis risk perception (Kennedy et al. 2022; Leos-Toro et al., 2020; Park et al. 2022; Salloum et al. 2018). Risk perception is shaped by a complex interaction of individual and environmental factors, such as use experience, individual differences, and contextual components, with knowledge of risks playing a key role in this process (Cheng et al. 2017; Jenkins et al. 2024; Park et al. 2022; Salloum et al. 2018). Increased risk knowledge has been linked to decreased current use among young people, while decreased knowledge has been linked to greater intention to use in the future (Harrison et al. 2024). Several studies from the US and Canada found that while most young adults report a general awareness of adverse physical or mental health effects of cannabis use, more specific risks, such as developing dependence, anxiety issues or psychosis, for instance, are less commonly known (Kennedy et al. 2022; Leos-Toro et al. 2020; Park et al. 2020, 2022, 2023; Salloum et al. 2018). The situation in Europe is less explored, but similar findings have been reported (Martínez-Vispo & Dias, 2022). Few studies have investigated cross-national differences in cannabis risk knowledge, yet it has been found that knowledge levels are higher among people in countries or states in which cannabis has been legalised compared to those living under more strict cannabis regulations (Goodman and Hammond 2022).

To educate young adults adequately, it is essential to understand in which areas of information young adults might lack knowledge (Martínez-Vispo & Dias, 2022). As many health behaviours manifest during early adulthood (Daw et al. 2017), imparting adequate cannabis health knowledge among young adults could allow for long-term safer use practices and reduce ill-informed decision-making towards cannabis use.

The current study explored: (1) gaps in knowledge of risks across different health dimensions among young adults in Canada, Germany, and the UK and how knowledge levels might differ between countries and cannabis use status, and (2) associations between sociodemographic and use-related variables and knowledge level. To explore differences in risk knowledge at country level, three countries with varying cannabis policies and use prevalences were included in this study. In Canada, recreational use has been legal for adults since 2018 (Health Canada, 2025). In Germany, at the time of data collection the legalisation of recreational use was anticipated, yet had not entered into force (Manthey et al. 2023). In the UK, recreational cannabis use is currently not legal (Home Office, 2024).

## Methods

Data are cross-sectional findings from the 2023 International Cannabis Policy Study (ICPS) conducted in Canada, the United States, Australia, New Zealand, the United Kingdom, and Germany (Hammond et al. 2023). Data were collected via self-completed web-based surveys conducted in September and October 2023 with respondents aged 16–65 years. The analyses included a subsample of participants aged 18–25 years living in Canada, Germany, or the UK at the time of data collection, to assess cannabis health knowledge among young adults across countries with varying cannabis policies.

A full description of the study methods can be found in the ICPS technical report (Iraniparast et al. 2023) and a methodology paper (Hammond et al. 2020). Respondents provided informed consent prior to completing the survey. The study was reviewed by and received ethics clearance through the University of Waterloo Research Ethics Committee (ORE#31330).

### Measures

#### Sociodemographic

Sociodemographic information included participants’ country, age, gender identity, income adequacy (i.e. how easy or difficult it is to financially meet needs) and student status. Participants’ gender identity was categorised into female, male and diverse (Gogovor et al. 2021). Student status was coded into a dichotomous variable (i.e. being currently enrolled at school or university or not).

#### Cannabis use status

Past-year cannabis use was categorised into one of three use statuses: regular consumers (weekly or (almost) daily use), occasional consumers (used within past 12 months, up to monthly use) and abstainers (never used or no use within past 12 months). Problematic cannabis use was measured by adopting items from the Alcohol, Smoking and Substance Involvement Screening Test (ASSIST) of the World Health Organisation (WHO, 2002). Participants were then categorised into being at low, moderate, or high risk of harm based on their ASSIST score (range 0–39), with a score of 0–3 indicating low risk and 4–26 indicating moderate risk. A score of 27 or more indicated high risk of harm from use and risk of dependence or acute dependence.

Peer use status (i.e. how many of the five closest friends of the participant use cannabis) was coded into a dichotomous variable (i.e. do any of their five closest friends use? – Yes or No/Don’t know).

#### Items assessing risk knowledge

**Fig. 1:**
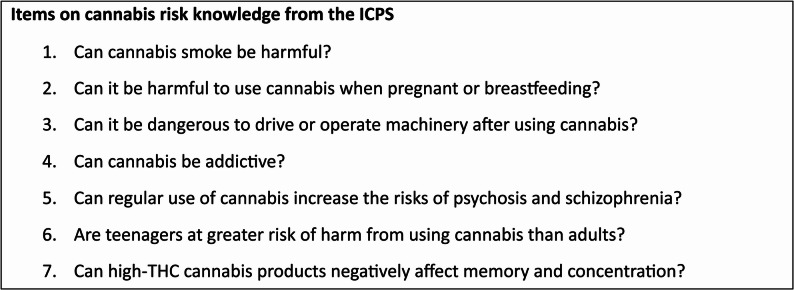
Items listed in the ICPS, based on the Cannabis Act of the Canadian government (2019). Response options included Yes, Maybe, No, Don’t know and Refuse to answer. For all items, the correct response is Yes

Questionnaire items on risk knowledge were based on the proposed warnings on cannabis use listed in the Cannabis Act by the Canadian government (2019) and covered different health-related dimensions (Fig. 1). Participants were asked to answer seven items based on what they knew or believed, selecting between *Yes*, *Maybe*, *No*, *Don’t know* or *Refuse to answer*. For the descriptive analyses, *Maybe* and *Don’t know* were categorised as an ambivalent response. For parts of the inferential analyses, incorrect (*No* for all items) and ambivalent responses were categorised as inaccurate, as they indicated that participants were not certain about the risks associated with cannabis use and therefore demonstrated an absence of knowledge. *Refuse to answer* was treated as missing data, and observations with missing predictor or outcome values were taken out of the regression models. For the regression analyses, an index variable was constructed by counting the number of inaccurate responses (i.e. incorrect and ambivalent responses) for each participant to assess risk knowledge level. This score ranged from 0 to 7, with a score closer to 0 indicating a higher knowledge level due to having fewer inaccurate responses, and a score closer to 7 indicating a lower knowledge level due to having more inaccurate responses.

### Data analysis

Data were weighted with post-stratification sample weights constructed using a raking algorithm to calibrate to known population proportions of age group, gender, region, school status, and cannabis use status (in Germany and the UK) from the census and other national surveys in each country (further information can be found in Iraniparast et al. 2023). Weights were rescaled to fit the final sample size.

Descriptive analyses were conducted to assess sociodemographic data, cannabis use, and inaccurate responses to items on risk knowledge across all three countries. For each item, the prevalence of inaccurate responses was investigated to assess the proportion of participants with low risk knowledge level between countries and use status. In inferential analyses, Rao-Scott design-adjusted chi-square tests, reported as design-based F statistics, were used to assess whether differences in the prevalence rates of inaccurate responses varied significantly between countries and use status.

Negative binomial regression was run to test for associations of several sociodemographic and cannabis use-related factors with risk knowledge level. To investigate differences in knowledge levels between abstainers, occasional consumers and regular consumers, separate analyses were run. In all models, risk knowledge level was the criterion while country, age, gender identity, school status, income adequacy, and ASSIST score (for occasional and regular consumers only) comprised the predictors. Negative binomial regression analyses were conducted due to non-normality (positive skew) and overdispersion of the criterion across the entire sample and when stratified by use status. Findings were reported as incidence rate ratios (IRR) with 95% confidence intervals and at an alpha level of 5%. For all analyses, weighted data were used. Analyses were conducted using Stata 15.1.

### Sample description

A total of 27,011 respondents completed the 2023 survey in Canada (*n* = 19,964), Germany (*n* = 3,603), and the UK (*n* = 3,444). After data cleaning, the analytic sample included 2,945 participants (60.5% female, mean age = 22, SD = 2.2). Inclusion criteria were being aged 18–25 years and living in Canada (*n* = 2,047), Germany (*n* = 446), or the UK (*n* = 452). Among the weighted subsamples, regular (16.8%) and occasional use (18.7%) were most prominent in Canada, whereas the UK had the lowest prevalence of regular (6.5%) and occasional consumers (10.9%), as well as the lowest prevalence of participants at moderate or high risk of harm from their use (39.2%, Table 1). Canada had the highest prevalence of participants at moderate or high risk of harm from use (46.9%).

**Table 1:** Participant characteristics and cannabis use status (*n* = 2,945)

		Total sample			UK			Germany			Canada		
		Unweighted (*n* = 2,945)		Weighted (*n* = 2,945)	Unweighted (*n* = 452)		Weighted (*n* = 385)	Unweighted (*n* = 446)		Weighted (*n* = 413)	Unweighted (*n* = 2,047)		Weighted (*n* = 2,147)
Age (years) (mean, SD)		22.0 (2.2)		21.7 (2.3)	21.8 (2.1)		21.7 (2.1)	22.2 (2.1)		21.9 (2.2)	22.0 (2.2)		21.7 (2.3)
		n (%)		n (%)	n (%)		n (%)	n (%)		n (%)	n (%)		n (%)
Gender													
	Male	1,120 (38.0)		1,361 (46.2)	192 (42.5)		191 (49.6)	232 (52.0)		203 (49.2)	696 (34.0)		967 (45.0)
	Female	1,783 (60.5)		1,547 (52.5)	254 (56.2)		192 (49.9)	204 (45.7)		201 (48.7)	1,325 (64.7)		1,155 (53.8)
	Gender-diverse	42 (1.4)		37 (1.3)	6 (1.3)		2 (0.5)	10 (2.2)		9 (2.2)	26 (1.3)		25 (1.2)
Income Adequacy^a^													
	(Very) difficult	893 (30.3)		929 (31.6)	127 (28.1)		117 (30.4)	104 (23.3)		91 (22.0)	662 (32.3)		720 (33.5)
	Neither easy nor difficult	1,028 (34.9)		1,019 (34.6)	150 (33.2)		129 (33.5)	182 (40.8)		176 (42.6)	696 (34.0)		715 (33.3)
	(Very) easy	889 (30.2)		838 (28.5)	161 (35.6)		119 (30.9)	147 (33.0)		126 (30.5)	581 (28.4)		594 (27.7)
Currently a student		1,651 (56.1)		1,762 (59.8)	224 (49.6)		190 (49.4)	224 (50.2)		238 (57.6)	1,203 (58.8)		1,334 (62.1)
Cannabis use status													
	Abstainers	1,620 (55.0)		2,032 (69.0)	159 (35.2)		318 (82.6)	221 (49.6)		329 (79.7)	1,240 (60.6)		1,385 (64.5)
	Occasional consumers	773 (26.3)		495 (16.8)	184 (40.7)		42 (10.9)	148 (33.2)		51 (12.4)	441 (21.5)		402 (18.7)
	Regular consumers	552 (18.7)		417 (14.2)	109 (24.1)		25 (6.5)	77 (17.3)		33 (8.0)	366 (17.9)		360 (16.8)
Peer use status													
	No friends who use	852 (28.9)		1,044 (35.5)	101 (22.4)		162 (42.1)	128 (28.7)		159 (38.5)	623 (30.4)		723 (33.7)
	1–5 friends who use	1,873 (63.6)		1,656 (56.2)	306 (67.7)		169 (43.9)	299 (67.0)		237 (57.4)	1,268 (61.9)		1,250 (58.2)
	Don’t know	196 (6.7)		222 (7.5)	41 (9.1)		52 (13.5)	16 (3.6)		16 (3.9)	139 (6.8)		154 (7.2)
Level of problematic use (ASSIST Score)^b^		*n* = 1,750	*n* = 1,421		*n* = 336	*n* = 161		*n* = 302	*n* = 213		*n* = 1,112	*n* = 1,077	
	Low risk	934 (53.4)		766 (53.9)	161 (47.9)		97 (60.3)	136 (45.0)		96 (45.1)	637 (57.3)		572 (53.1)
	Mid risk	650 (37.1)		542 (38.1)	126 (37.5)		50 (31.1)	136 (45.0)		73 (34.3)	388 (34.9)		419 (38.9)
	High risk	166 (9.5)		113 (8.0)	49 (14.6)		13 (8.1)	30 (9.9)		15 (7.0)	87 (7.8)		86 (8.0)

Country-related percentages refer to each country subsample. Due to rounding and the exclusion of missing values, figures in the table may not sum precisely to the stated totals

^a^How easy or difficult it is for the participant’s family to pay for the things they need

^b^*The Alcohol*,* Smoking and Substance Involvement Screening Test* (WHO, 2002) – Score calculated for cannabis use

## Results

### Prevalence of cannabis risk knowledge gaps

**Fig. 2:**
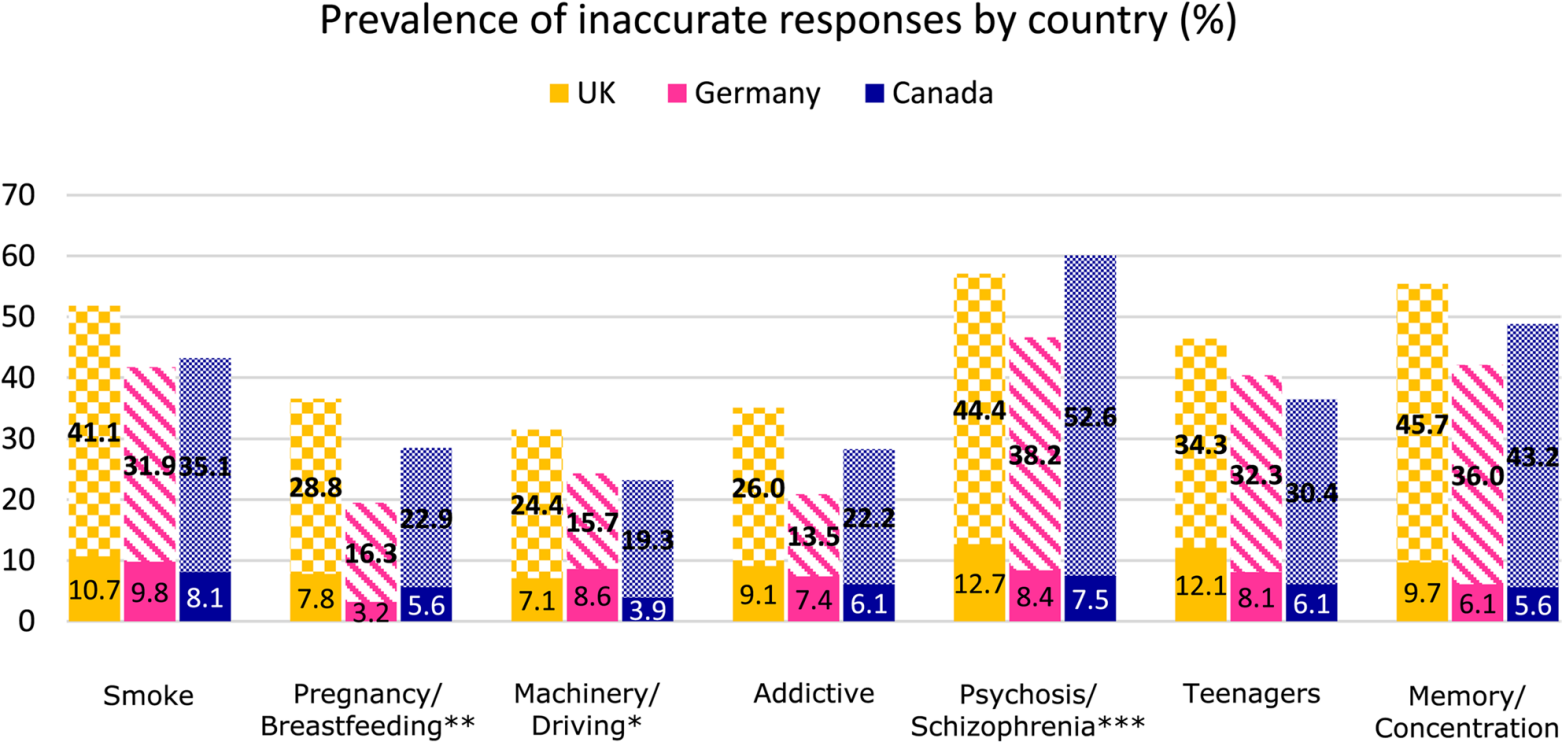
Weighted prevalence of inaccurate responses given by participants were measured across all seven cannabis-related health questions across the UK (*n* = 385), Germany (*n* = 413) and Canada (*n* = 2,147). Prevalence is presented as percentages. The solid sections of the graphs show incorrect responses while the patterned sections show ambivalent responses. Differences in prevalence across countries were tested for each drug using Rao-Scott design-adjusted chi-square tests, whereby *p < 0.05, **p < 0.01 and ***p < 0.001. Non-significant differences (*p* ≥ 0.05) are not marked

Young adults from the UK had the highest prevalence of incorrect or ambivalent responses for six out of the seven questions addressing risks of cannabis use, and Canadian participants for one (Fig. 2). German participants had the lowest prevalence of inaccurate responses for five items. Rao-Scott design-adjusted chi-square tests showed that prevalence rates of inaccurate responses differed significantly (*p* ≤ 0.05) between countries for items addressing pregnancy and breastfeeding (F = 2.92, *p* = 0.005), operating machinery (F = 2.25, *p* = 0.027) and risk of psychosis and schizophrenia (F = 4.08, *p* < 0.001), but not for any other items (Table 2).

**Table 2:** Results of Roa-Scott design-adjusted chi-square tests for risk items

Variables		Design-based F statistic
Country	Smoke	F(8, 22223) = 1.44, *p* = 0.177
	Pregnant/breastfeeding	F(7, 19868) = 2.92, *p* = 0.005
	Machinery	F(7, 20626) = 2.25, *p* = 0.027
	Addiction	F(7, 19886) = 1.97, *p* = 0.058
	Psychosis/schizophrenia	F(7, 21631) = 4.08, *p* < 0.001
	Teenagers	F(7, 21482) = 1.73, *p* = 0.094
	Memory/concentration	F(8, 22679) = 1.95, *p* = 0.052
Use Status	Smoke	F(8, 22381) = 3.23, *p* = 0.001
	Pregnant/breastfeeding	F(7, 21808) = 3.57, *p* < 0.001
	Machinery	F(7, 20562) = 3.71, *p* < 0.001
	Addiction	F(8, 21923) = 5.43, *p* < 0.001
	Psychosis/schizophrenia	F(8, 21996) = 2.58, *p* = 0.010
	Teenagers	F(7, 21918) = 1.48, *p* = 0.165
	Memory/concentration	F(8, 23079) = 5.53, *p* < 0.001

Results are reported as design-based F statistics. All data were weighted

The level of significance was *p* ≤ 0.05

Items with the highest prevalence of inaccurate responses (i.e. incorrect or ambivalent responses) across all three countries were those addressing risk of psychosis and schizophrenia (46.6% to 60.1%), followed by the questions on memory and concentration (42.1% to 55.4%) and harm from cannabis smoke (41.7% to 51.8%).

**Fig. 3:**
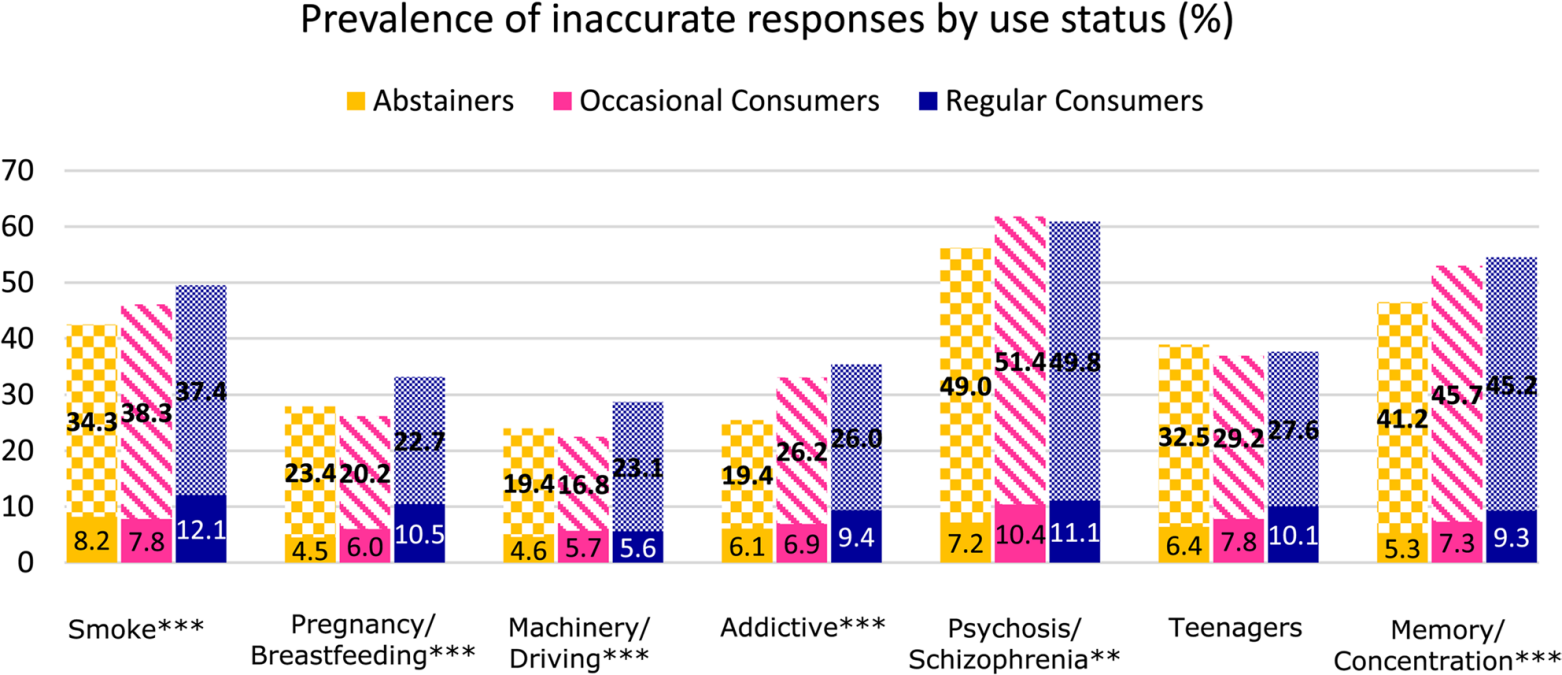
*The weighted total sample (n = 2*,*945) was stratified by use status*,* with the categories abstainers (n = 2*,*032)*,* occasional consumers (n = 495) and regular consumers (n = 417)*,* before the weighted prevalence of inaccurate responses among each subgroup was measured for all cannabis-related health questions. The solid sections of the graphs show incorrect responses while the patterned sections show ambivalent responses. Differences in prevalence across countries were tested for each drug using Rao-Scott design-adjusted chi-square tests*, whereby *p < 0.05, **p < 0.01 and ***p < 0.001. Non-significant differences (p ≥ 0.05) are not marked

Between participants with different use statuses, within the entire sample (*n* = 2,945), regular consumers had a slightly higher prevalence of inaccurate responses compared to occasional consumers and abstainers for five out of seven items (Fig. 3). This difference was significant for all items except the item addressing increased risk of harm to teenagers (Table 2). Similar to the country comparison, the items with the highest prevalence of inaccurate responses among all consumer groups were addressing psychosis and schizophrenia (56.2% to 61.8%), memory and concentration (46.5% to 54.5%) and harm from smoke (42.5% to 49.5%). There was a significant association between risk knowledge level (i.e. number of inaccurate responses) and use status (F = 1.86, *p* = 0.027).

### Associations with knowledge level across use status

For the entire sample, male participants (IRR = 1.138, CI = 1.047–1.237) had a 13.8% higher rate of inaccurate responses compared to female participants (Table 3). Compared to being from Canada, being from the UK (IRR = 1.177, CI = 1.038–1.335) was associated with a 17.7% higher rate of inaccurate responses, while being from Germany (IRR = 0.866, CI = 0.758–0.989) was associated with a 13.4% lower rate. Participants who reported regular use (IRR = 1.162, Cl = 1.038–1.301) had a 16.2% higher rate of inaccurate responses compared to abstainers.

**Table 3:** Parameter estimates for the negative binomial regression analyses of risk knowledge level

Effect		Total sample (*n* = 2,715)					Abstainers (*n* = 1,469)				Occasional Consumers (*n* = 730)					Regular Consumers (*n* = 516)			
		IRR	*p*		95% CI for IRR		IRR	*p*	95% CI for IRR		IRR	*p*	95% CI for IRR			IRR	*p*	95% CI for IRR	
				*LB*		*UB*			*LB*	*UB*				*LB*	*UB*			*LB*	*UB*
Gender																			
	Female (ref.)																		
	Diverse	1.201	0.366	0.808		1.785	1.449	0.127	0.900	2.333	0.812	0.554		0.407	1.620	0.853	0.694	0.386	1.885
	Male	1.138	**0.002**	1.047		1.237	1.116	**0.044**	1.003	1.242	1.222	**0.028**		1.022	1.460	1.058	0.552	0.879	1.273
Age		1.017	0.075	0.998		1.037	1.015	0.249	0.990	1.040	1.041	**0.036**		1.003	1.080	0.988	0.595	0.945	1.033
Country																			
	Canada (ref.)																		
	UK	1.177	**0.011**	1.038		1.335	1.154	0.072	0.987	1.349	1.165	0.063		0.992	1.368	1.227	**0.020**	1.033	1.457
	Germany	0.866	**0.033**	0.758		0.989	0.800	**0.011**	0.674	0.949	1.006	0.948		0.848	1.193	1.096	0.557	0.807	1.490
Use status																			
	Abstainers (ref.)																		
	Occasional consumers	1.085	0.110	0.982		1.199	-	-	-	-	-	-		-	-	-	-	-	-
	Regular consumers	1.162	**0.009**	1.038		1.301	-	-	-	-	-	-		-	-	-	-	-	-
Risk of harm (ASSIST)																			
	Low risk (ref.)																		
	Mid risk	-	-	-		-	-	-	-	-	1.236	**0.025**		1.028	1.487	0.701	**0.001**	0.572	0.860
	High risk	-	-	-		-	-	-	-	-	1.818	**< 0.001**		1.405	2.351	0.869	0.301	0.665	1.135
Peer use status																			
	No (ref.)																		
	Yes	0.991	0.846	0.903		1.088	1.034	0.534	0.930	1.150	0.718	**0.001**		0.594	0.868	1.047	0.808	0.724	1.514
Income adequacy																			
	Neither easy nor difficult (ref.)																		
	(Very) difficult	0.936	0.187	0.849		1.033	0.932	0.288	0.817	1.062	0.885	0.203		0.734	1.068	1.022	0.839	0.831	1.255
	(Very) easy	0.937	0.188	0.850		1.033	0.935	0.286	0.827	1.058	0.848	0.092		0.701	1.027	1.055	0.660	0.831	1.339
Student status																			
	No (ref.)																		
	Yes	0.965	0.409	0.890		1.050	0.928	0.187	0.830	1.037	1.115	0.167		0.955	1.302	1.016	0.863	0.852	1.211

Stratified by use status. All data included in the analyses were weighted. Final sample sizes are reported, after removal of observations with missing predictor or outcome values. The level of significance was *p* ≤ 0.05

#### Stratification by use status

Among regular consumers, being from the UK (IRR = 1.227, CI = 1.033–1.457) was associated with a 22.7% higher rate of inaccurate responses compared to being from Canada. Participants at moderate risk of harm from their use (IRR = 0.701, CI = 0.572–0.860) had a 29.9% lower rate compared to those at low risk.

Among occasional consumers, with every additional year of age (IRR = 1.041, CI = 1.003–1.080), the rate of inaccurate responses increased by 4.1%. Male participants (IRR = 1.222, CI = 1.022–1.460) had a 22.2% higher rate of inaccurate responses compared to their female peers. Participants at moderate risk of harm from their use (IRR = 1.236, CI = 1.028–1.487) had a 23.6% higher rate, and those at high risk (IRR = 1.818, CI = 1.405–2.351) had an 81.8% higher rate of inaccurate responses compared to those at low risk. Participants with at least one close friend who uses cannabis (IRR = 0.718, CI = 0.594–0.868) had a 28.2% lower rate of inaccurate responses compared to those without use among friends.

Among abstainers, being male (IRR = 1.116, CI = 1.003–1.242) was associated with a 11.6% higher rate of inaccurate responses, compared to being female. German participants (IRR = 0.800, CI = 0.674–0.949) had a 20.0% decreased rate of inaccurate responses compared to being Canadian.

## Discussion

This study investigated cannabis risk knowledge gaps among young adults in Canada, Germany, and the UK across different health dimensions, both at the country and the individual level. Associations between sociodemographic and use-related factors and risk knowledge levels were explored.

Descriptive and inferential analyses showed some evidence for cross-national differences in risk knowledge levels, yet those differences were not significant across all health dimensions. Regression analyses showed that young adults in the UK were less and those in Germany more knowledgeable compared to Canadians. The difference in knowledge levels between the UK and Canada supports previous findings on increased knowledge levels in areas with liberal cannabis policies compared to countries with stricter regulations (Goodman and Hammond 2022). Although there seems to be no direct relationship between risk perception and cannabis policies (Lemos et al. 2023), legalisation of recreational use could allow for an increase in risk awareness among the population through public education campaigns and health warning labels (Goodman et al. 2022). In Germany, at the time of data collection, the legalisation of recreational cannabis use was impending, causing an ongoing public debate on cannabis use led by political parties, professional associations, and the cannabis industry (Manthey 2023). This might have exposed more young adults to cannabis-related information and could explain heightened knowledge levels despite strict cannabis policy, even in comparison to Canadian participants. However, differences in cannabis policies and their public health responses merely provide context for these results, allowing for speculation, but not conclusions about causal relationships. While results of the regression analyses showed an overall difference in knowledge levels between countries, item-level analyses indicate that these differences cannot be generalised across all aspects of cannabis health knowledge but rather depend on specific risks. Besides cannabis policy, differences in knowledge levels across countries could reflect sociocultural differences, meaning the associations and belief systems young adults in different cultures hold towards cannabis use (Heath 2001; Rafei et al. 2023), to which dissimilarity in quality and quantity of preventive efforts might contribute (Sumnall 2022). Differences in participants’ characteristics on country-level could play into use patterns, which could in turn lower risk perceptions (Pessar et al. 2024). At the individual level, findings showed an association between cannabis use patterns of the participants and risk knowledge level. While these findings complement the literature on how use frequency is linked to decreased risk knowledge and risk perception (Kennedy et al. 2022; Leos-Toro et al., 2020; Park et al. 2022; Salloum et al. 2018), results offer a novel perspective on the interplay of experiencing harm from use and use frequency in risk knowledge levels among young cannabis consumers. Being at moderate to high risk of harm as an occasional consumer was associated with decreased risk knowledge, yet moderate risk of harm paired with regular use increased knowledge levels. It has been found that general cannabis risk perception among people aged 50 or older is higher among those with cannabis use disorder (Choi, DiNitto & Marti, 2018), yet this study did not investigate knowledge of risks. In addition, being at moderate risk of harm as measured by the ASSIST tool does not indicate having a cannabis use disorder (WHO,  2002), and being at high risk of harm as a regular consumer was not associated with knowledge level. The types of products consumed by participants could play into general risk perception levels: Young adults’ risk perception has been found to vary between routes of administration and level of potency of the cannabis products consumed, especially regarding regular consumption (Leos-Toro et al. 2020). Young adults who regularly consume higher-potency cannabis products (e.g. oils or tinctures) might have an overall greater risk perception compared to those who occasionally consume lower-potency alternatives, such as smoking dried herbs, despite having a higher use frequency. However, this dimension was not accounted for in the analyses and future studies are needed to investigate this perspective further. Although it remains unclear why risk knowledge level changed in relation to problematic use, these findings indicate differences in risk knowledge among consumers experiencing harm from their use and underline a need for future research to understand possible windows of opportunity for risk education in this high-risk group.

Besides use status, other sociodemographic factors were associated with participants’ knowledge of risks. Gender differences in knowledge levels were found, with male participants with no or occasional use being less knowledgeable than their female peers, which aligns with other research (Harris-Lane et al. 2023). When educating young adults on risks of cannabis use, young men might be less open to accepting risk information due to having more confidence in knowledge compared to young women, even if their beliefs are not objectively correct (Park et al. 2022). Although results of the current study did not show to what extent young men specifically were certain or ambivalent about their responses, considering gender differences in knowledge levels could be important to effectively reach young adults of all gender identities. More research is needed to explore these differences in detail.

Older occasional consumers in this study showed decreased risk knowledge levels compared to their younger peers; however, this could be rooted in growing use experience with increased age, rather than age-specific factors (Park et al. 2022; Salloum et al. 2018). Having a friend who consumes cannabis increased risk knowledge in occasional consumers, which might partly be explained by peers playing a role in the communication of information on cannabis among young adults (Cheng et al. 2017; Park et al. 2020), yet it could also stem from exposure to negative aspects of cannabis consumption among friends. Together, these findings highlight a need for future research on sociodemographic associations with cannabis risk knowledge levels to understand how different sociodemographic circumstances of young adults might shape their relationship to cannabis use.

Although overall risk knowledge levels varied between participants from different countries and with different cannabis use statuses, the relative level of knowledge across most health dimensions was similar. In line with previous findings (Leos-Toro et al., 2020; Martínez-Vispo & Dias, 2022), more specific risks, such as increased risk of psychosis and schizophrenia through regular use or the effect of specifically high-THC products on memory and concentration, were less well known compared to more general risks, such as “potential danger” from driving or operating machinery after use. Possibly, developing a severe health condition, such as psychosis, at some point in the future seems generally less likely to a young person, than, for instance, having a traffic accident (i.e. immediate vs. delayed risk, Larsman et al. 2012), making a long-term risk more difficult to remember than a risk more relevant to their everyday life. Differences in risk knowledge across health items could lie in general awareness of adverse health outcomes in the population and association with self, as, for instance, risk during pregnancy or traffic accidents are issues that affect greater proportions of people than psychosis and schizophrenia (Goodman and Hammond 2022). In terms of education on risks, these results provide specific information on potential knowledge gaps in young adults, which could be helpful in tailoring prevention programmes towards this target group.

This study is subject to limitations common to survey research, whereby ICPS-specific limitations have been discussed previously (Iraniparast et al. 2023). Despite the use of post-stratification weights, results are based on a non-probability sample and therefore cannot be interpreted as strictly representative of the population. By grouping uncertain and incorrect responses in the negative binomial regressions, the analyses capture overall absence of correct knowledge but do not distinguish between those theoretically distinct response types. Future research should investigate uncertainty of risks and being misinformed separately to allow for a more nuanced understanding of factors associated with absence of knowledge of cannabis health risks. Overall, the degree of ambivalence among participants who did not know of the risks of cannabis use was high, which could be interpreted as increased risk perception, yet this can only be speculated on. This study did not investigate to what extent participants were exposed to cannabis-related health information prior to participation, which could further explain differences in risk knowledge levels across all three countries and inform preventative practice.

## Conclusion

This study demonstrates that there are differences in the level of knowledge of risks associated with cannabis among young adults at the individual and at the country level. However, the same set of risks is less well known across countries and young adults with different use experience, indicating that certain subject areas may universally be underrepresented in the education of young people on the risks of cannabis use, or that young adults across different contexts attribute less relevance to certain topics than others. To empower young adults to make informed decisions on cannabis consumption, interventions should implement a detail-focused education of all risks associated with cannabis use, while taking contextual factors among this target group into consideration.

## Data Availability

The ICPS Survey can be found here: https://cannabisproject.ca/wp-content/uploads/2023/10/ICPS-ENGLISH-SURVEY-W6-2023-Master-Oct-26-CLEAN.pdf. The datasets used and/or analysed during the current study are available from Prof. David Hammond upon reasonable request.
